# Dopaminergic modulation of emotional conflict in Parkinson's disease

**DOI:** 10.3389/fnagi.2014.00164

**Published:** 2014-07-23

**Authors:** Vanessa Fleury, Emilie Cousin, Virginie Czernecki, Emmanuelle Schmitt, Eugénie Lhommée, Antoine Poncet, Valérie Fraix, Irène Troprès, Pierre Pollak, Alexandre Krainik, Paul Krack

**Affiliations:** ^1^Department of Neurology, Geneva University HospitalGeneva, Switzerland; ^2^Movement Disorder Unit, Department of Psychiatry and Neurology, Grenoble University HospitalGrenoble, France; ^3^Psychology and Neurocognition Laboratory, UMR CNRS 5105, Pierre Mendès-France UniversityGrenoble, France; ^4^Unit 610, Federation of Nervous System Disease, National Institute of Health and Medical Research (INSERM), Pitié-Salpêtrière HospitalParis, France; ^5^Department of Health and Community Medicine, Geneva University HospitalGeneva, Switzerland; ^6^Joseph Fourier University – Grenoble IGrenoble, France; ^7^Grenoble Neuroscience Institute, INSERM-UJF-CEA-CHU U836Grenoble, France; ^8^IRMaGe, Université Grenoble AlpesGrenoble, France; ^9^US 017, INSERMGrenoble, France; ^10^UMS 3552, CNRSGrenoble, France; ^11^Neuroradiology and MRI, Grenoble University HospitalGrenoble, France

**Keywords:** emotional stroop, dopamine, Parkinson's disease, cingulate cortex, non-motor fluctuations

## Abstract

Neuropsychiatric fluctuations in Parkinson's disease (PD) are frequent and disabling. One way to investigate them is to assess the ability to inhibit distractive emotional information by a modified emotional Stroop (ES) task. We compared non-depressed, non-demented PD patients with healthy controls. During an acute levodopa challenge, patients performed a modified ES task during functional MRI and a neuropsychological assessment including Visual Analog Mood (VAMS) and Apathy scales. Ten patients and 12 controls completed the study. The VAMS scores were significantly improved by the acute intake of levodopa (*p* = 0.02), as was the apathy score (*p* = 0.03). Negative ES task (i.e. fearful facial expressions with the words “happy” or “fear” written across them), induced a lengthening of the mean reaction time during the incongruent trials compared with the congruent trials in controls (relative difference = 2.7%, *p* < 0.001) and in ON patients (relative difference = 5.9%, *p* < 0.001), but not in OFF patients (relative difference = 1.7%, *p* = 0.28). Controls and ON patients displayed greater activation than OFF patients within the right pregenual anterior cingulate cortex (pACC), an area specifically involved in emotional conflict resolution (*p* < 0.001 and *p* < 0.008 respectively, *k* > 5 uncorrected). No difference in the activation of the pACC was found between controls and ON patients, suggesting a normalization of the activation following levodopa administration. These results suggest that emotional conflict processes could be dopamine-dependent. Pregenual ACC hypoactivation could be directly due to the degeneration of dopaminergic mesocorticolimbic pathway. Our results propose that neuropsychiatric fluctuations in PD patients could be partially explained by pACC hypoactivation and that adjustments of dopaminergic medication might be helpful for their treatment.

## Introduction

Although non-motor fluctuations (NMF) occur frequently throughout the course of Parkinson's disease, their prevalence is often underestimated. They can be more disabling than motor symptoms. NMF frequently manifest in relationship to motor fluctuations (Witjas et al., 2002) and fluctuate in parallel with them (Storch et al., 2013). Forms of NMF include neuropsychiatric, dysautonomic, and sensory symptoms (Riley and Lang, 1993). Neuropsychiatric fluctuations encompass apathy, anxiety, sadness, slowness of thinking, fatigue during drug-off condition, and mood elation with euphoria and increased alertness in drug-on state (Witjas et al., 2002; Fox and Lang, 2008; Lhommée et al., 2012). Previous publications have demonstrated that mood and anxiety fluctuations are related to levo-dopa dosing (Friedenberg and Cummings, 1989; Maricle et al., 1995b; Richard et al., 2005). Different mechanisms have been proposed to explain neuropsychiatric fluctuations such as mesocorticolimbic dopaminergic denervation underlying off-period related neuropsychiatric symptoms (Thobois et al., 2010), sensitization related to severity of the mesocorticolimbic lesion in combination with pulsatile dopaminergic treatment with levodopa (Voon et al., 2011; Castrioto et al., 2013) and/or relatively selected stimulation of mesocorticolimbic dopamine D3 receptors with dopamine agonists (Thobois et al., 2013). Both mood and anxiety disorders have a high prevalence among parkinsonian patients (Reijnders et al., 2008; Dissanayaka et al., 2010). Certain authors suggest that their impact on quality of life is more important than the impact of motor signs (Schrag, 2006; Chaudhuri and Schapira, 2009). In the “NMF in Parkinson's disease study,” anxious and depressive symptoms were highly associated with drug-off state. Beck Depression Inventory (BDI) scores, obtained while parkinsonian patients were on chronic dopaminergic medication, only correlated with depression severity in drug-off but not in drug-on state, suggesting that depression scoring in fluctuating patients mainly reflects the mood in drug-off state during NMF (Storch et al., 2013). Consequently, a better understanding of neuropsychiatric fluctuations physiopathology seems necessary in order to improve parkinsonian patient's mood symptoms management and quality of life.

Patients with depression or anxiety disorders show a higher sensitivity toward negatively valenced stimuli, which results in slower processing of negative emotional information (Williams and Nulty, 1986; Becker et al., 2001). A method for investigating inhibition of distractive emotional information (i.e. emotional interference) is the emotional Stroop (ES) task (Williams et al., 1996). Variants of the ES task have been developed such as the word-face Stroop task (Stenberg et al., 1998) or the modified ES task (Etkin et al., 2006). In this latter task, subjects are asked to identify the affect of faces with fearful or happy expressions while ignoring the words “happy” or “fear” written across them. Words are either congruent or incongruent with faces. Emotional conflict arises when the word is not in agreement with the facial expression. It induces an emotional interference resulting in a decision-making slowdown with slower reaction time to name the affect of faces if the emotion of the word written across the face is incongruent with the facial expression. Serra-Mestres and Ring (2002) demonstrated that non-depressed parkinsonian patients presented with significantly greater emotional interference than did controls, despite matching for depression score, supporting the hypothesis that non-depressed parkinsonian patients were more vulnerable to the interfering effects of negative emotional stimuli. This vulnerability could be a factor contributing to the increased risk of developing depression in parkinsonian patients, because in the presence of sadness, patients will remain in that state for longer periods.

Neuroimaging studies suggested that the pregenual part of the anterior cingulate cortex (ACC), also known as the rostral anterior cingulate cortex (rACC), plays a major role in ES task performance (Whalen et al., 1998; Etkin et al., 2006; Egner et al., 2008). The rACC is located in the medial surface of the brain and is anterior to the genu of the corpus callosum. It is a key structure for the integration of emotion and cognition (Pessoa, 2008). It is considered to be a part of the affective subdivision of the ACC (together with the subgenual ACC) because it has connections with the limbic and paralimbic regions, such as the amygdala, the orbitofrontal cortex (Devinsky et al., 1995) and the nucleus accumbens (Johansen-Berg et al., 2008). The rostral ACC is specifically associated with the resolution of emotional conflict (i.e., overcoming of conflict), whereas the lateral prefrontal cortex resolves exclusively non-emotional conflict (Egner et al., 2008). Conversely, a common area of the dorsal ACC, which is located in the posterior region of this structure, detects and signals the occurrence of conflict in information processing (Botvinick et al., 1999; Kerns et al., 2004). This region is activated by both emotional and non-emotional conflict monitoring (Egner et al., 2008).

The neurotransmission involved in ES processes is largely unknown. The ACC is involved in the regulation of attention (Posner and DiGirolamo, 1998). As it has been suggested that dopaminergic signals serve to draw attention to salient events of all sorts (Gray et al., 1997) and that dopamine is a critical component involved in the attribution of salience of attractive or aversive valence to stimuli (Bressan and Crippa, 2005), it is possible that dopamine plays a role in ES processes. Parkinson's disease is primarily due to degeneration of dopaminergic neurons and offers the opportunity to investigate the pharmacological manipulation of the dopaminergic system in humans. Abnormal behavioral performances to the ES task have been found in Parkinson's disease (Serra-Mestres and Ring, 1999, 2002) and in psychiatric conditions such as cocaine or heroin addiction in which dopaminergic dysfunction is well demonstrated (Marissen et al., 2006).

We hypothesized that dopamine would play a role in emotional interference processes and that mood disturbances during neuropsychiatric fluctuations in parkinsonian patients could be explained by an abnormal rACC functioning. We assessed fluctuating non-depressed parkinsonian patients during their drug-on and drug-off states while performing a modified ES task during functional MRI (fMRI), in order to better understand the pathophysiology of neuropsychiatric fluctuations.

## Materials and methods

### Participants

Patients were recruited from the Grenoble University Hospital Movement Disorders Clinic. The selection criteria were as follows: presence of clinically diagnosed Parkinson's disease according to the United Kingdom Parkinson's Disease Society Brain Bank clinical criteria for Parkinson's disease (Hughes et al., 1992), presence of motor and non-motor fluctuations, age < 70 years, the ability to tolerate the experimental period in the MRI machine during a drug-off state (i.e., ≥11 h after the patient's last dopaminergic drug dose the night before) and during a drug-on state (i.e., 1 h after a dose of levodopa which efficiently controls parkinsonian symptoms without disabling dyskinesia) and the absence of a contraindication to an MRI. The patients were compared to healthy volunteers who were recruited from the community. Healthy controls (HC) had no history of neurological or psychiatric disorders. They were chosen to match the patient group as closely as possible for age, sex, and education level.

Exclusion criteria for both groups were: the presence of dementia as indicated by a score of ≤130 on the Mattis Dementia Rating Scale (DRS) (Mattis, 1976; Schmidt et al., 1994) or a score ≤12 on the Frontal Assessment Battery (FAB) (Dubois et al., 2000; Kaszás et al., 2012), the presence of moderate to severe depression as indicated by a score ≥20 on the BDI-II (Beck et al., 1996), the presence of psychosis, and/or the presence of impaired face recognition as indicated by a score < 41 on the long-form of the Benton Recognition Test (Levin et al., 1975; Benton, 1994). Subjects with fMRI-related issues (i.e. unsatisfactory picture quality caused by participants moving within the scanner during acquisition or other scanner-specific technical complications) were excluded. The Ethics Committee of the Grenoble University Hospital approved the study. After a complete description of the study, the participants gave written informed consent in accordance with the Grenoble University Hospital Review Board guidelines and the Declaration of Helsinki.

### Procedures

Each participant completed a modified ES task during fMRI followed by a neuropsychological assessment. Each parkinsonian patient was studied twice in a counterbalanced manner during a levodopa challenge where the drug-off and the drug-on states were performed on two consecutive mornings. The drug-off state followed an overnight withdrawal of all antiparkinsonian drugs. The drug-on state was performed in a fasting state in order to improve levodopa absorption and to enable more precise timing of the maximum therapeutic effect. Levodopa was administrated orally using a levodopa-benserazide dispersible formulation. For each patient, the dose of levodopa used for the challenge was calculated as 100% of his levodopa equivalent first morning dose. The equivalent dose was calculated according to Tomlinson et al. (2010). We decided against the use of suprathreshold doses of levodopa (i.e., 130–150% of the levodopa equivalent first morning dose) in order to not prime dyskinesias, which could have induced fMRI movement artifacts, and to avoid modifying cognition. Before each MRI scan, all patients underwent a neurological examination to rate the severity of their motor function using the Unified Parkinson's Disease Rating Scale (UPDRS) motor rating items 18–31 in drug-on and drug-off conditions (Fahn and Elton, 1987). Levodopa and dopamine agonist doses were expressed as total levodopa-equivalent daily dosage (LEDD) (Tomlinson et al., 2010). All participants were asked to refrain from nicotine and caffeine for ≥4 h prior to the fMRI studies.

#### fMRI stimuli

**Figure 1:**
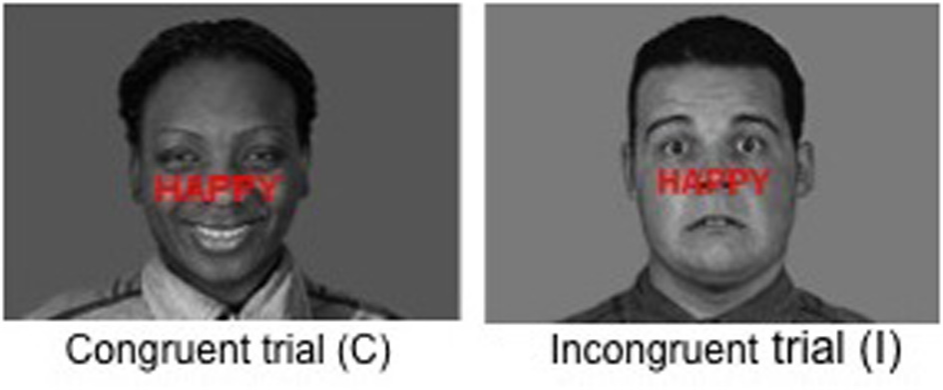
**Examples of stimuli used in the modified emotional Stroop task**.

The modified ES task consisted of 136 presentations of black and white happy and fearful facial expression photographs drawn from the Montreal Set of Facial Displays of Emotion (Beaupré and Hess, 2005). This battery consists of emotional facial expressions by men and women of European, Asian, Hispanic and African decent. Each expression had been coded according to the Facial Action Coding System (Ekman and Friesen, 1978) to assure identical expressions across actors. The words “FEAR” or “HAPPY” were prominently written in red bold “Arial” size 45, centered on the middle of faces (i.e., on the “nose” position). The face–word association created either a congruent condition (32 happy faces with the word “HAPPY,” 32 fearful faces with the word “FEAR”) or an incongruent condition (32 happy faces with the word “FEAR,” 32 fearful faces with the word “HAPPY”) (Figure 1). Eight additional faces in the incongruent condition (happy faces with the word “FEAR”) were added, in order to obtain 136 presentations to allow the construction of a pseudoaleatory design with blocks of 17 pictures presented 8 times. All faces were resized to 1024 × 717 pixels and size was controlled by e-prime2 software (100% of the initial size). Gender, ethnic group (Caucasian, Asian, African and Hispanic), and facial expressions (fear and happiness) were counterbalanced across conditions (congruent or incongruent) and across trial sequences. There were neither direct repetitions of the same face with varying word distracters, in order to avoid negative priming effects, nor direct repetitions of exact face-word-distracter combination, in order to avoid repetition priming effects (Etkin et al., 2006). All conditions (i.e., congruent and incongruent) were displayed in pseudorandom order.

#### Emotional stroop task

Participants were instructed to judge as fast and as accurately as possible whether facial expressions represented fear or happiness while ignoring the word. After receiving instructions, participants underwent a short training session immediately prior to the experiment, with items that were not shown during the real experiment.

**Figure 2:**
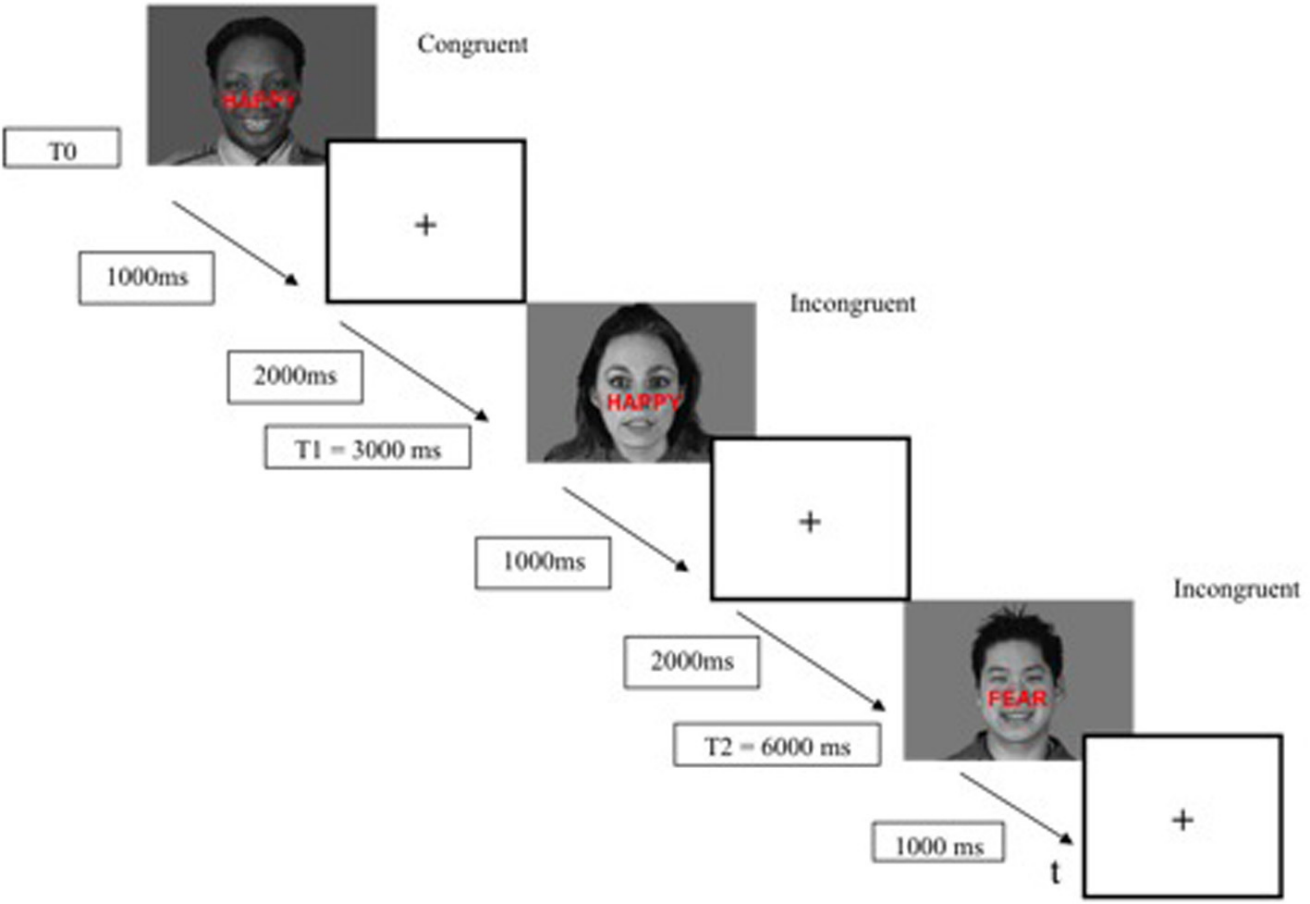
**Example timeline used in the modified emotional Stroop task fMRI paradigm**.

The ES task was presented with the E-Prime 2 software (E-Prime Psychology Software Tools Inc., Pittsburgh, USA) and was displayed on a back-projection screen that was viewed by the subjects via a mirror attached to the head-coil. Each trial began with a stimulus (face-word association) displayed for 1000 ms, followed by a fixation cross for 2000 ms (interstimulus interval of 3000 ms) (Figure 2). The responses (*fear* and *happy*) were recorded by means of two response keys pressed by the index finger of the subject's dominant hand. The mean response time and percentage of correct responses were recorded for each participant. The duration of the ES task was 7 min.

#### fMRI data acquisition

BOLD fMRI data were collected while subjects performed the ES task in a 3Tesla Bruker Medspec S300 MRI scanner using a gradient–echo T2^*^–weighted Echo Planar Imaging (EPI) sequence covering 39 axial, interleaved slices (3.2 mm thick, 0 mm gap), beginning at the cerebral vertex and encompassing the entire cerebrum and the cerebellum (time repetition (TR) = 2500 ms; time echo (TE) = 30 ms; flip angle = 77°; field of view (FOV) = 216 × 216 mm^2^; matrix = 72 × 72; voxel size = 3 × 3 × 3.2 mm). Six dummy scans were done before image acquisition to avoid the effects of signal saturation. All scanning parameters were selected to optimize the quality of the BOLD signal while maintaining a sufficient number of slices to acquire whole-brain data. For structural whole-brain images, three-dimensional T1–weighted sagittal images of the whole-brain were also acquired (TR = 2500 ms; TE = 4.3 ms; TI = 908.1 ms; flip angle = 8°; FOV = 256 × 224 × 176 mm^3^; voxel size = 1.33 × 1.75 × 1.37 mm^3^).

#### Neuropsychological assessment

All participants performed a neuropsychological evaluation including the Mattis DRS for global cognitive assessment, the FAB for dysexecutive dysfunction assessment, the BDI for depression assessment, the State-Trait Anxiety Inventory for Adults (Spielberger, 1983) to assess their anxiety level and the Benton Recognition Test for facial perception assessment. Patients continued their standard dopaminergic medication during this evaluation. The presence and the intensity of NMF were evaluated with the Ardouin Scale (Ardouin et al., 2009). The NMF, which patients experienced in daily life on their usual treatment, were assessed by adding the two items “drug-on NMF” and “drug-off NMF.” A score ≥ 1 was taken as indicative of the presence of NMF.

During their drug-off and drug-on states, participants performed a second neuropsychological assessment in a counterbalanced manner, comprising a Visual Analog Mood Scale (VAMS) (Norris, 1971) to measure the acute levodopa effects on patient's subjective states, an Starkstein Apathy Scale (SAS) where a score ≥ 14/42 corresponded to the diagnosis of apathy (Starkstein et al., 1992), and an Ekman Facial Affect Test (Ekman and Friesen, 1976) to assess the ability to identify facial expressions of emotions (happiness, sadness, fear, disgust, surprise, anger, neutral).

### Statistical analysis

#### Statistical analysis of behavioral data

Accuracy (% of correct responses) and reaction time (RT) latencies were recorded during the fMRI experiment. Mean task accuracy was calculated as the average percentage of trials correctly identified relative to the sum of trials. Error trials (wrong answers and omissions) were excluded from the RT analysis. Different conditions were distinguished: incongruent trials with negative or positive faces and congruent trials with negative or positive faces. RT was log transformed to normalize the data. Differences in performances between groups (HC, drug-on and drug-off patients), congruency (congruent or incongruent) and emotion (negative or positive faces) were assessed using linear mixed effects models with a random effect on subject. Interaction terms between congruency and emotion, and between congruency and group were introduced in the model. ES effect was defined as a lengthening of the mean RT during the incongruent trials compared with the congruent trials. Statistical significance was assessed at the 0.05 level for all analyses.

#### fMRI statistical analysis

Data analysis was performed by using the general linear model as implemented in SPM8 (Wellcome Department of Imaging Neuroscience, London, UK) where each event is modeled using a hemodynamic function model. Data analysis started with several spatial pre-processing steps. First, the functional volumes were time-corrected with the second slice as reference. Subsequently, all volumes were realigned to correct head motion using rigid body transformations. After discarding the four first slices, the first volume of the fMRI session was taken as the reference volume. To correct for interaction between head movements and EPI distortions, unwarping was performed by using the individually acquired fieldmaps (Andersson et al., 2001). Weighted anatomical volume was co-registered to mean images created by the realignment procedure and was normalized to the Montreal Neurological Institute (MNI) space. The anatomical segmentation parameters were subsequently used for the normalization of anatomical and functional volumes. Finally, each functional volume was smoothed by an 8-mm Full Width at Half Maximum Gaussian kernel to ameliorate differences in intersubject localization. Time series for each voxel were high-pass filtered (1/128 Hz cutoff) to remove low-frequency noise and signal drift.

With regards to congruence [Congruence (C) vs. Incongruence (I)] and emotion [Positive for facial expression of happiness (P), Negative for facial expression of fear (N)] for each participant, we defined four experimental conditions (CP, CN, IP, IN). These conditions were modeled as four regressors and convolved with the canonical form of the hemodynamic response function. Moreover, movement parameters derived from realignment corrections (3 translations and 3 rotations) were also taken into account in the design matrix to remove the movement-related variance.

Next, several statistical analyses were performed. At the first level analysis (within-group comparisons), the following effects were evaluated: the ES effect with all faces (I vs. C) [I (P+N) vs. C (P+N)] as well as the ES effect with only the positive faces analyzed [I (P) vs. C (P)] and the ES effect with only the negative faces analyzed [I (N) vs. C (N)] (i.e., Negative ES contrast), using a one-sample *t*-test, in order to identify cerebral regions involved in emotional conflict. At the second level analysis (between-group level), we performed a random-effect between-group analysis by using paired *t*-tests (Friston et al., 1998) according to the Negative ES contrast defined at the individual level analysis in order to characterize cerebral differences between parkinsonian patients in their drug-off and drug-on states, and HC.

## Results

Twelve parkinsonian patients and twelve HC were included in the study. One patient was not able to complete the SE task in the MRI machine during his drug-off state because of a painful dystonia and was excluded from all analyses. Another patient was excluded because of unsatisfactory picture quality caused by participant movements during image acquisition in the MRI. Consequently, data from ten patients (seven males, age range: 51–66 years, mean age: 60, *SD*: 4.2) and twelve HC (8 males, age range: 46–69 years, mean age: 60.1, *SD*: 6.4) were included in the analyses. Clinical characteristics of each group are reported in Table 1.

**Table 1:** **Demographic and clinical characteristics of participants**.

	**Patients *n* = 10)**	**Controls *n* = 12)**	***p*-value**
Age (years)	60 ± 4.2	60.1 ± 6.4	ns
Sex ratio (male:female)	7:3	8:4	
Education duration (years)	10.7 ± 5	12.4 ± 5.2	ns
Disease duration (years)	9 ± 3.1		
Levodopa-equivalent daily dose (mg/day)	917.7 ± 367.6		
Drug-off UPDRS motor score (/108)	33.8 ± 10.8		
Drug-on UPDRS motor score(/108)	11.4 ± 7.4^*^		
Non-motor fluctuation Ardouin score (/8)	2.1 ± 0.9		
Mattis DRS score^**^ (/144)	138.2 ± 4.5	141.2 ± 2.0	ns
FAB^**^ (/18)	15.8 ± 1.8	17.3 ± 0.9	0.03
Benton^**^ (/54)	47.3 ± 2.3	47.6 ± 2.5	ns
BDI^**^ (/63)	11.5 ± 7.9	5.4 ± 5.5	0.04
STAI state^**^ (/80)	34 ± 17.1	24.5 ± 4.4	ns
STAI trait^**^ (/80)	37.6 ± 8.4	32.75 ± 6.8	ns

*Values are expressed as mean (±SD) scores. BDI, beck depression inventory; FAB, frontal assessment battery; Mattis DRS, dementia rating scale; n = number of participants; ns = non-significant; STAI, State-Trait Anxiety Inventory for Adults; UPDRS motor score, motor scale of Unified Parkinson's Disease Rating Scale*.

*p significant if <0.05 when compared with controls (Student test for age and education level; Mann-Whitney test for score on the Mattis, FAB, Benton, BDI, STAI tests)*.

* *p = 0.005 for difference between the drug-off and drug-on UPDRS motor scores (Wilcoxon's test for paired data)*.

** *Scores obtained from patients on chronic dopaminergic medication*.

All patients were on dopamine replacement medications. Patients and HC did not statistically differ in terms of age, education, gender distribution, global cognitive performance, facial perception or anxiety level. The parkinsonian group scored significantly lower on the FAB score than the controls (*p* = 0.03), although the averaged score fell within the non-pathological range (>14/18). Patients scored significantly higher on the BDI score than the controls (*p* = 0.04), although the averaged score fell within the non-depressed range (<12/63). In terms of psychotropic medications, two patients were taking antidepressant drugs (sertraline or mirtazapine) and four were taking benzodiazepines (clonazepam or prazepam). The psychotropic drug dosage was low and had been stable for at least 6 months in all patients. None of the control subjects was on psychotropic medication.

### Neuropsychological results during the levodopa challenge

The results are presented in Table 2.

**Table 2:** **Neuropsychological characteristics of patients during the levodopa-challenge**.

	**Group 1**	**Group 2**	***p*-Value**
	**Drug-off patients Mean ± *SD***	**Drug-on patients Mean ± *SD***	**Group 1 vs. Group 2**
SAS (/42)	13 ± 2.6	9.5 ± 3.6	0.03
Norris VAS			
Asthenia	42.3 ± 14.2	12.2 ± 11.2	0.02
subscore (/80)			
Affective	25.7 ± 16.3	11.8 ± 8.6	0.02
subscore (/80)			
**EKMAN FAT (% CR)**			
Happiness	100 ± 0	97.1 ± 9.0	ns
Fear	52.8 ± 17.9	64.3 ± 28.7	0.08
Surprise	90 ± 13.6	91.4 ± 10	ns
Sadness	72.84 ± 23.8	62.8 ± 21.5	ns
Disgust	80 ± 23.5	77.1 ± 20.5	ns
Anger	61.4 ± 27	78.6 ± 22.6	ns
Neutral	81.4 ± 16.6	78.6 ± 31	ns

*Values are expressed as mean (±SD) scores. Ekman FAT, Ekman Facial Affect test; ns, non-significant; SAS, Starkstein Apathy Scale; VAS, Visual Analog Mood Scale; %CR, percentage of correct response. p significant if <0.05 (Wilcoxon test for paired data for the intra-group comparisons)*.

In patients, apathy, as measured by the SAS, significantly improved with L-dopa intake between drug-off and drug-on conditions (*p* = 0.03). The affective and asthenia subscores of the VAMS were significantly improved by the acute intake of levodopa (*p* = 0.02). In the drug-off condition, patients had a tendency to suffer from a selective impairment in facial emotion recognition of fear compared with drug-on patients (*p* = 0.08) while there was no difference in correct responses for other emotional expressions.

### Behavioral data

**Figure 3:**
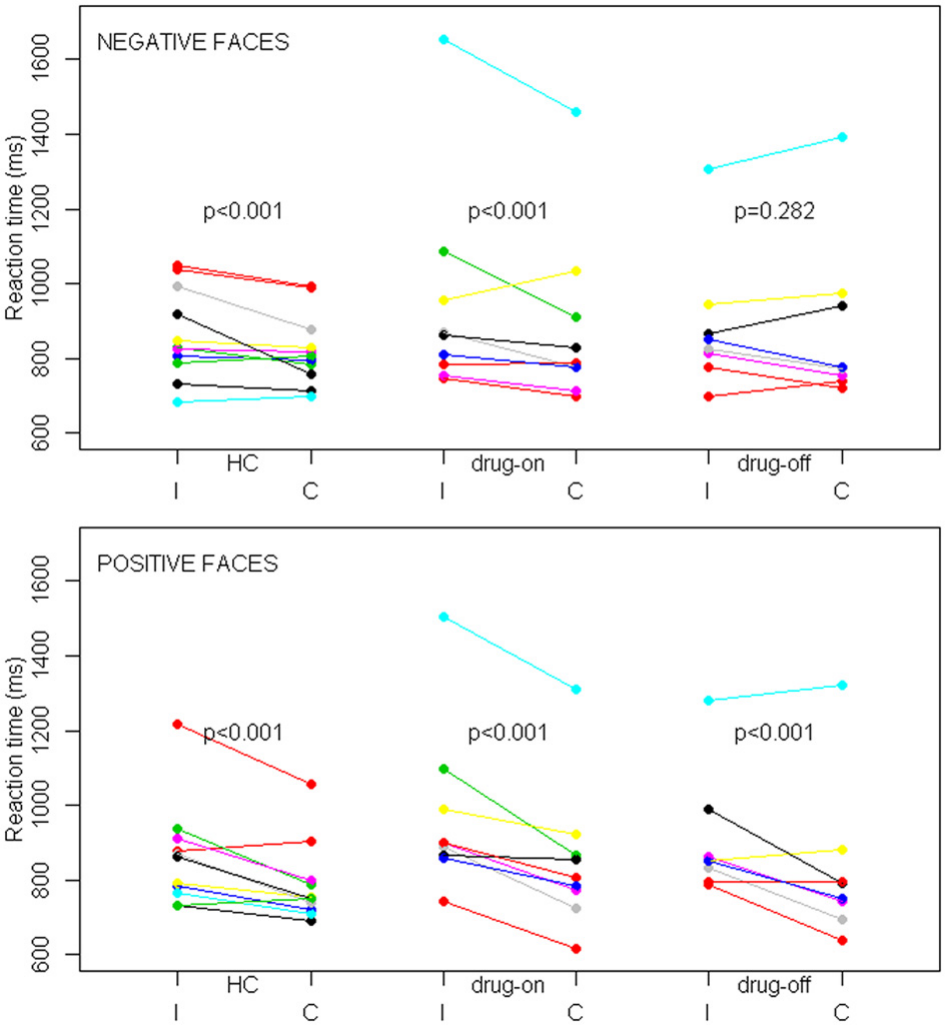
**SE task behavioral results in controls (HC), drug-off and drug-on parkinsonian patients**. Data are patients' mean reaction times expressed in milliseconds (ms) depending on the congruence (I, incongruent; C, congruent) and facial emotion conditions among the different groups. The top panel represents trials with negative faces and the bottom panel represents trials with positive faces. *p* represents the statistical significance of the reaction time difference between incongruent and congruent conditions. Differences were assessed with a linear mixed effects model.

The percentage of correct responses and RT were unavailable for two drug-off patients, one drug-on patient and one healthy control, due to technical difficulties during MRI. The mean task accuracy was not statistically different between the three groups with an overall accuracy of 80%. The percentage of correct responses was higher during congruent trials than during incongruent trials, regardless of the group (*p* = 0.002). Facial emotions did not influence the percentage of correct responses. The reaction time results are presented in Figure 3. In trials with positive faces, our multivariate mixed effect model indicated a significant effect of congruency on performances in each group (*p* < 0.001 for all three groups), with a shorter RT in the congruent condition compared with the incongruent condition (in HC, drug-on and drug-off patients, the mean relative decrease in RT was 8.6% [95%CI: 6.2–11.0], 11.7% [95%CI: 9.1–14.2] and 7.7% [95%CI: 4.9–11.4], respectively). In trials with negative faces, there was a significant effect of congruency on performances in HC (relative difference = 2.7% [95%CI: 0–5.3], *p* < 0.001) and drug-on patients (relative difference = 5.9% [95%CI: 3.1–8.7], *p* < 0.001). No significant effect of congruency was found in the drug-off patients in trials with negative faces (relative difference = 1.7% [95%CI: -1.4–4.6], *p* = 0.282). The congruence effect was not statistically different between HC and the drug-on or drug-off patients, whereas it was significantly larger for the drug-on patients compared to the drug-off patients (*p* = 0.02), regardless of the facial emotion.

### fMRI within-group comparisons

#### ES task validation in healthy participants

**Figure 4:**
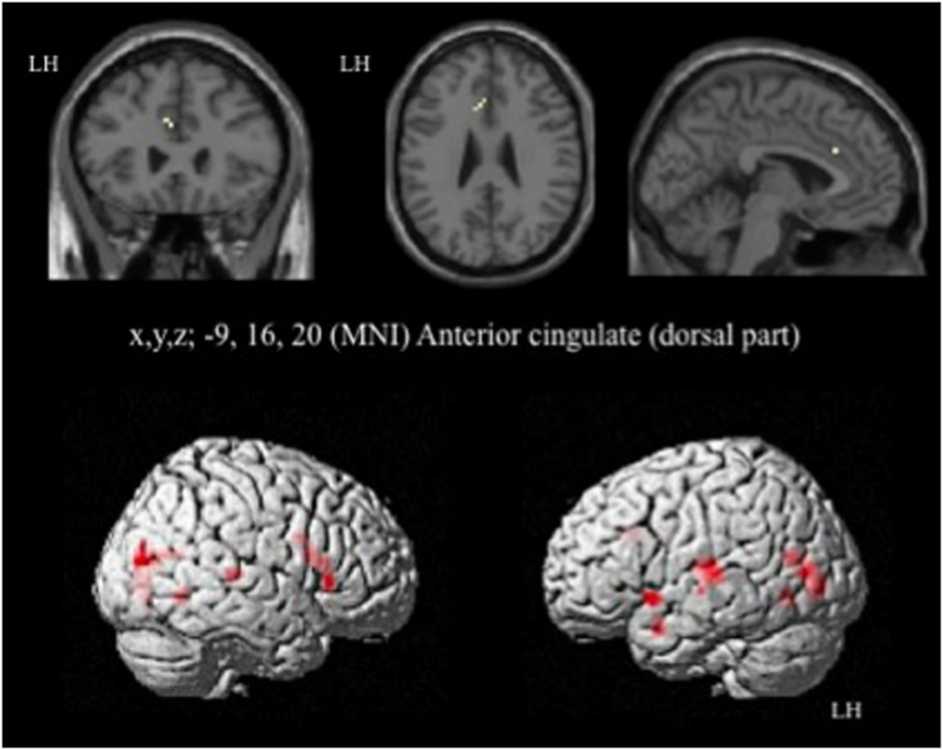
**Brain activation during the emotional Stroop task (contrast: Incongruent vs. Congruent with all faces) in healthy subjects**. *p* < 0.001, *k* > 5, *n* = 12, uncorrected. LH, left hemisphere; MNI, Montreal Neurological Institute.

In HC, the network of cerebral activation elicited by the Incongruent vs. Congruent [I-C] contrast is shown in Figure 4 and Table 3. A significant activation within the postgenual ACC (x, y, z MNI coordinates: −9, 16, 20; *p* < 0.001; *k* > 5 uncorrected) was identified. No significant activation was found in the rACC or in the amygdala, areas specifically activated in emotional conflict.

**Table 3:** **Brain regions activated during the emotional Stroop task using all faces in healthy subjects**.

**Contrast**	**Cerebral areas**	**H**	**BA**	**MNI coordinates (x, y, z)**	**k**	***T***
[I-C] (all faces)	**Cingulate cortex**					
	Anterior cingulate	L	24	−9, 16, 20	9	4.95
	**Frontal cortex**					
	Inferior frontal triangularis	R	45	54, 23, 4	8	4.74
	**Temporal cortex**					
	Fusiform	L	19	−24, −64, −2	9	5
	Superior temporal	L	41	−51, −22, 8	49	7.82
		R	41	51, −28, 8	4.75	6
	Superior temporal pole	L	13	−27, 8, 21		
		L	38	−54, 8, −8	10	4.69
	Middle temporal	L	19	−45, −64, 14	10	4.69
	Inferior temporal	R	37	39, −58, −5	6	4.71
	**Insula**	R	47	33, 26, 1	32	6.1
	**Occipital cortex**					
	Middle occipital	L	19	−45, −76, 8	5	6.31
	Lingual	L	17	−24, −76, 11	49	8.27
	Calcarine	R	19	36, −82, 14	41	6.88
		R	18	15, −79, 1	11	4.60
		L	18	−6, −70, 14	22	6.83
[I-C] (all faces)	*No suprathreshold voxels*					

*Cerebral activation locations refer to maximal hemodynamic response sites. p < 0.001, k > 5, n = 12, uncorrected. Cerebral areas are defined by the Automatic Anatomical Labeling. BA, Brodmann areas; C, congruent; H, hemisphere; I, incongruent; k, cluster size (number of voxels); L/R, left/right; MNI, Montreal Neurological Institute; x, y, z, mediolateral, rostrocaudal, and dorsoventral*.

**Figure 5:**
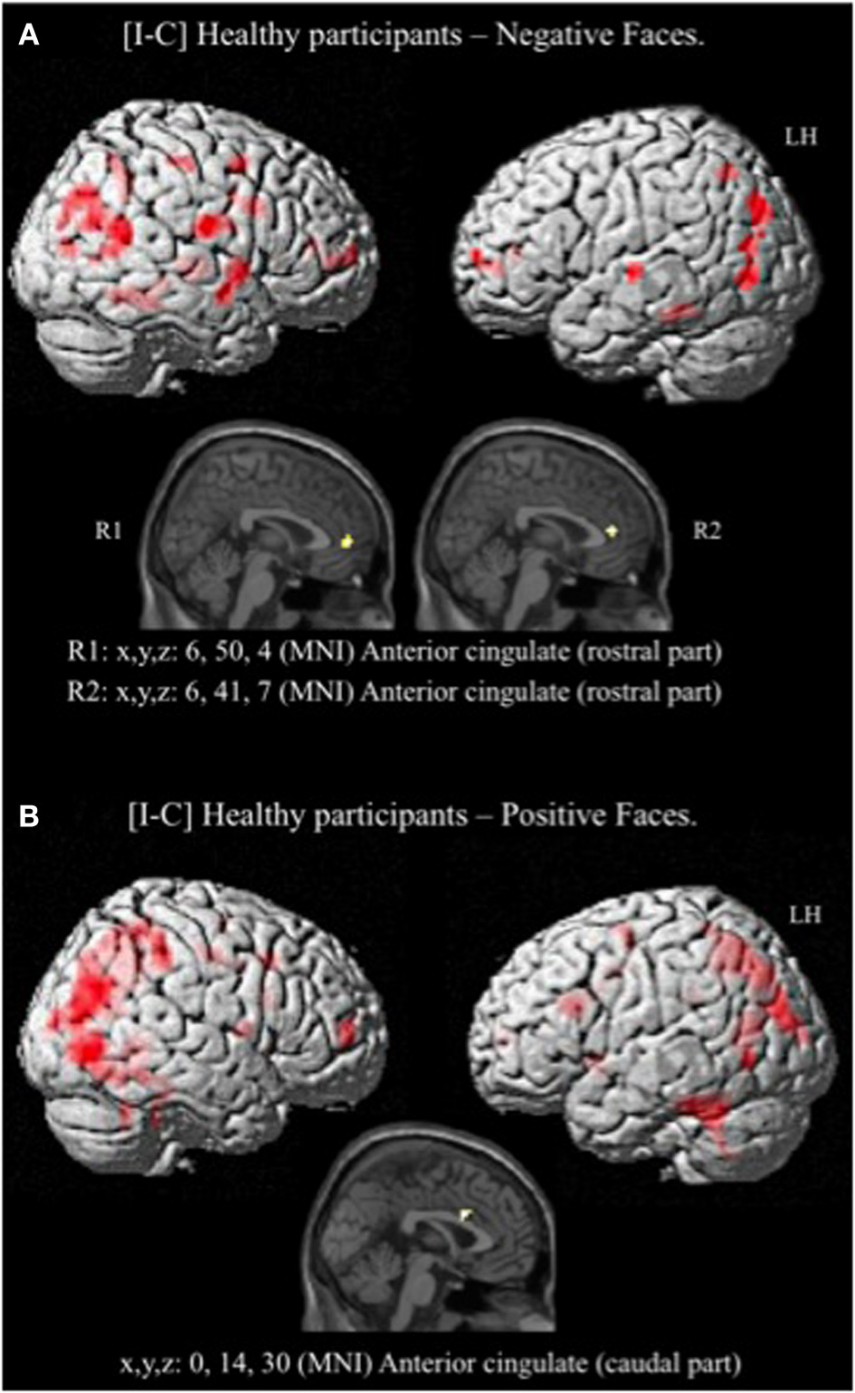
**Brain activation during the emotional Stroop task (contrast: Incongruent vs. Congruent) with separate analysis of negative (A) and positive (B) faces, in healthy subjects**. *p* < 0.001, *k* > 5, *n* = 12, uncorrected. C, congruent; I, incongruent; LH, left hemisphere; MNI, Montreal Neurological Institute; R1, region 1; R2, region 2; x, y, z: mediolateral, rostrocaudal, and dorsoventral.

Given that no significant activation was found in the rACC, and because numerous studies have reported rACC activation during “sad” ES tasks (i.e., classic ES task using negatively valenced words written in different ink colors) (Whalen et al., 1998, for review), we decided to decorrelate the analysis depending on the emotion of the face. For the contrast [I-C] for negative faces, the rACC was consistently activated. Two regions, R1 and R2 were activated within this area (R1: x, y, z MNI coordinates: 6, 50, 4; Brodmann area (BA) = 32; *p* < 0.001; *k* > 5 uncorrected, and R2: x, y, z: 6, 41, 7; *BA* = 24; *p* < 0.001; *k* > 5 uncorrected) (Figure 5A and Table 4). Other areas less specifically involved in emotional conflict were also activated, such as the superior parietal gyrus and the superior temporal gyrus, which has been involved in the perception of emotion in facial stimuli. The right inferior frontal gyrus, which has been typically implicated in go/no go tasks (Aron et al., 2004), was engaged. The amygdala was not activated.

**Table 4:** **Brain regions activated during the emotional Stroop task when only negative faces were analyzed in healthy subjects**.

**Contrast**	**Cerebral areas**	**H**	**BA**	**MNI coordinates (x, y, z)**	**k**	***T***
[I-C] (negative faces)	**Cingulate cortex**					
	Anterior cingulate	L	32	6, 50, 4	55	6.73
		R	24	6, 41, 7	11	5.46
	**Frontal cortex**					
	Inferior frontal opercularis	R	9	36, 11, 30	17	4.56
	Middle frontal	R	6	33, 2, 49	9	4.70
	**Parietal cortex**					
	Superior parietal	L	7	−18, −64, 52	13	4.80
	Postcentral	R	3	45, −22, 52	8	4.90
	Precuneus	R	30	15, −52, 11	18	5.09
	**Temporal cortex**					
	Fusiform	L	20	−36, −37, −18	17	5.34
		R	20	27, −37, −18	50	6.70
	Superior temporal	L	22	−63, −16, 1	7	8.48
	**Insula**	R	13	45, 5, −2	37	6.49
	**Thalamus**	R		15, −16, 1	51	8.64
	**Occipital cortex**					
	Middle occipital	L	19	−27, −76, 33	42	9.01
	Middle occipital	L	18	−42, −73, 1	30	8.86
	Middle occipital	R	39	51, −52, 14	146	7.76
[C-I] (negative faces)	*No suprathreshold voxels*					

*p < 0.001, k > 5, n = 12, uncorrected. Cerebral areas are defined by the Automatic Anatomical Labeling. BA, Brodmann areas; C, congruent; H, hemisphere; I, incongruent; k, cluster size (number of voxels); L/R, left/right; MNI, Montreal Neurological Institute; x, y, z, mediolateral, rostrocaudal, and dorsoventral*.

For the contrast [I-C] for positive faces, a significant activation within the caudal ACC was found (x, y, z: 0, 14, 30; *BA* = 24; *p* < 0.001; *k* > 5 uncorrected) but not in the rACC (Figure 5B and Table 5). The contrast [I-C] for positive faces also activated other areas commonly activated in non-emotional conflict (Egner et al., 2008), such as the anterior prefrontal cortex, the precuneus, the lingual gyrus and the inferior parietal gyrus. No suprathreshold voxels were activated for [C-I] contrast for positive, negative or all faces.

**Table 5:** **Brain regions activated during the emotional Stroop task when only positive faces are analyzed in healthy subjects**.

**Contrast**	**Cerebral areas**	**H**	**BA**	**MNI coordinates (x, y, z)**	**k**	***T***
[I-C] (positive faces)	**Cingulate cortex**					
	Anterior cingulate	L	24	0, 14, 30	31	5.49
	Middle cingulate	L	24	0, −1, 43	13	4.60
	**Frontal cortex**					
	Superior frontal	R	10	21, 56, 8	34	6.63
	**Parietal cortex**					
	Inferior parietal	R	7	27, −49, 56	61	5.34
	Precuneus	L	19	33, −70, 27	862	8.85
	**Temporal cortex**					
	Fusiform	L	37	−36, −46, −28	80	7.29
	Middle temporal	L	37	−48, −64, −2	7	5.19
	Inferior temporal	R	37	51, −43, −15	9	4.98
	**Basal ganglia**					
	Caudate nucleus	L		−12, 5, −5	13	5.70
	Pallidum	L		−12, 5, −5	13	5.70
	Putamen	L		−12, 5, −5	13	5.70
		R		36, 5, 11	8	4.96
	**Occipital cortex**					
	Superior occipital	L	18	−12, −91, 11	9	5.97
	Middle occipital	L	19	−36, −82, 24	36	6.58
	Lingual	R	19	15, −49, −2	84	7.51
	Calcarine	L	31	−12, −64, 17	60	8.40
	**Cerebellum**	L		−18, −52, −21	18	5.14
		R		27, −31, −21	15	4.70
[C-I] (positive faces)	*No suprathreshold voxels*					

*p < 0.001, k > 5, n = 12, uncorrected. Cerebral areas are defined by the Automatic Anatomical Labeling. BA, Brodmann areas; C, congruent; H, hemisphere; I, incongruent; k, cluster size (number of voxels); L/R, left/right; MNI, Montreal Neurological Institute; x, y, z, mediolateral, rostrocaudal, and dorsoventral*.

#### ES task in parkinsonian patients

Considering the results obtained in HC, we decided to study the ES effect with the contrast [I-C] for negative faces, as this was only contrast that activated the rACC. The cerebral sites of hemodynamic response during the ES task are shown in Table 6 for the drug-off patients and in Table 7 for the drug-on patients. Both patient groups activated less cerebral areas and recruited a decreased number of voxels for all activated brain areas compared to controls. Drug-off patients exhibited additional areas of activation not seen in HC and drug-on patients, such as the left superior medial frontal gyrus (MNI coordinates *x* = −3, *y* = 32, *z* = 46; *BA* = 8; *p* < 0.001, *k* > 5), the left hippocampus (*x* = −24, *y* = −37, *z* = −8; *p* < 0.001, *k* > 5), and the right cuneus (*x* = 12, *y* = −94, *z* = 14; *p* < 0.001, *k* > 5). No suprathreshold voxels were activated for [C-I] contrast for negative faces in any patients groups.

**Table 6:** **Brain regions activated during the emotional Stroop task with negative faces in drug-off patients**.

**Contrast**	**Cerebral areas**	**H**	**BA**	**MNI coordinates (x, y, z)**	**k**	***T***
[I-C] (negative faces)	**Frontal cortex**					
	Superior medial frontal	L	8	−3, 32, 46	10	5.82
	**Temporal cortex**					
	Inferior temporal	L	37	−51, −55, −8	7	6.02
	Hippocampus	L	28	−24, −37, −8	15	6.28
	**Occipital lobe**					
	Middle occipital	R	19	42, −79, 14	11	8.32
		R	19	30, −85, 20	7	5.09
	Cuneus	R	18	12, −94, 14	8	4.98
[C-I] (negative faces)	*No suprathreshold voxels*					

*p < 0.001, k > 5, n = 10, uncorrected. Cerebral areas are defined by the Automatic Anatomical Labeling. BA, Brodmann areas; C, congruent; H, hemisphere; I, incongruent; k, cluster size (number of voxels); L/R, left/right; MNI, Montreal Neurological Institute; x, y, z, mediolateral, rostrocaudal, and dorsoventral*.

**Table 7:** **Brain regions activated during the emotional Stroop task with negative faces in drug-on parkinsonian patients**.

**Contrast**	**Cerebral areas**	**H**	**BA**	**MNI coordinates (x, y, z)**	**k**	***T***
[I-C] (negative faces)	**Frontal cortex**					
	Middle frontal	R	46	45, 38, 17	5	5.05
	**Temporal cortex**					
	Middle temporal	L	22	−63, −31, 4	5	4.98
	Inferior temporal	R	19	39, −64, −8	13	7.71
[C-I] (negative faces)	*No suprathreshold voxels*					

*p < 0.001, k > 5, n = 10, uncorrected. Cerebral areas are defined by the Automatic Anatomical Labeling. BA, Brodmann areas; C, congruent; H, hemisphere; I, incongruent; k, cluster size (number of voxels); L/R, left/right; MNI, Montreal Neurological Institute; x, y, z, mediolateral, rostrocaudal, and dorsoventral*.

### fMRI between-group comparisons

**Figure 6:**
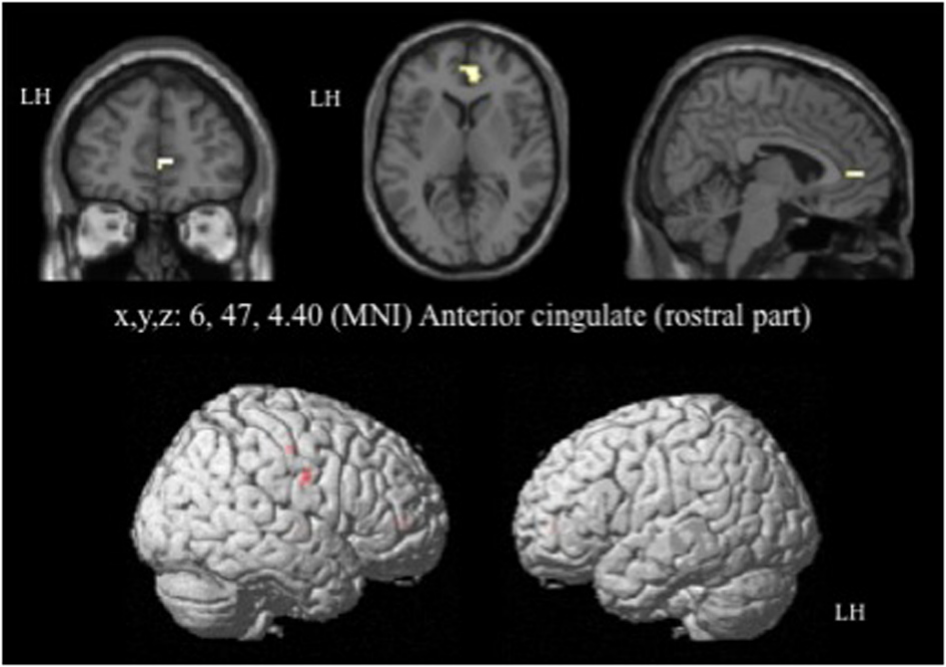
**Brain activation during the emotional Stroop task with negative faces (contrast [I-C]) in the between group comparison (Control vs. drug-off patients)**. *p* < 0.001, *k* > 5, *n*_healthy_ = 12, *n*_patients_ = 10, uncorrected. I; incongruent; C; congruent; LH, left hemisphere; x, y, z: mediolateral, rostrocaudal, and dorsoventral; MNI, Montreal Neurological Institute.

For the contrast [I-C] with negative faces, HC displayed significantly greater activation than off-drug patients within the right rACC (x, y, z: 6, 47, 4.4; *BA* = 32; *p* < 0.001; *k* > 5 uncorrected) (Figure 6), the right pre- and post-central gyri and the right thalamus (Table 8A). Drug-off patients vs. HC did not yield any suprathreshold clusters (Table 8B).

**Table 8:** **Comparisons between controls and drug-off patients: brain regions activated during the emotional Stroop task with negative faces**.

**Contrast**	**Cerebral areas**	**H**	**BA**	**MNI coordinates (x, y, z)**	**k**	***T***
[I-C] (negative faces)						
*A. Controls vs. Drug-off patients*						
	**Cingulate cortex**					
	Anterior cingulate	R	32	6, 47, 4.4	16	4.75
	**Frontal cortex**					
	Precentral	R	4	39, −16, 49	6	3.67
	**Parietal cortex**					
	Postcentral	R	6	54, −7, 30	5	3.78
	**Thalamus**	R		12, −10, 1	11	4.76
*B. Drug-off patients vs. Controls*						
	*No suprathreshold voxels*					
[C-I] (negative faces)						
*A. Controls vs. Drug-off patients*						
	*No suprathreshold voxels*					
*B. Drug-off patients vs. Controls*						
	*No suprathreshold voxels*					

*p < 0.001, k > 5, n_healthy_ = 12, n_patients_ = 10; uncorrected. Cerebral areas are defined by the Automatic Anatomical Labeling. BA, Brodmann areas; C, congruent; H, hemisphere; I, incongruent; k, cluster size (number of voxels); L/R, left/right; MNI, Montreal Neurological Institute; x, y, z, mediolateral, rostrocaudal, and dorsoventral*.

Comparisons between HC and drug-on patients (HC vs. drug-on patients and drug-on patients vs. HC) did not yield any suprathreshold clusters. Drug-on patients had significantly greater activation than drug-off patients in the right inferior temporal gyrus (x, y, z: 42, −61, −8; *BA* = 37; *p* < 0.001; *k* > 5 uncorrected), an area involved in visual recognition. No significant activation was found in the ACC for a *p* < 0.001. However, by reducing the threshold to *p* < 0.005, drug-on patients displayed greater activation than drug-off patients in the left postgenual ACC (x, y, z: −3, 23, 19; *BA* = 24; *T* = 4.28; *p* < 0.005; *k* > 5 uncorrected). For a *p* < 0.008, drug-on patients displayed greater activation than drug-off patients in the right rACC (x, y, z: 9, 38, 1; *BA* = 32; *p* < 0.008; *k* > 5 uncorrected) (Table 9). Drug-off patients did not exhibit any suprathreshold clusters when compared with drug-on patients at a *p* < 0.001 threshold. No activation was seen in the ACC when the threshold was increased to *p* < 0.008 (Table 9). No suprathreshold voxels were activated for [C-I] contrast for negative faces in any between-group comparisons.

**Table 9:** **Comparisons between drug-on and drug-off patients: cerebral sites of hemodynamic response during the emotional Stroop task with negative faces**.

**Contrast**	**Cerebral areas**	**H**	**BA**	**MNI coordinates (x, y, z)**	**k**	***T***
[I-C] (negative faces)						
*A. Drug-on vs. Drug-off patients*						
	**Temporal cortex**					
	Inferior temporal^*^	R	37	42, −61, −8	5	6.92
	**Cingulate cortex**					
	Dorsal anterior cingulate^**^	L	24	−3, 23, 19	21	4.28
	Rostral anterior cingulate^***^	R	32	9, 38, 1	5	3.59
*B. Drug-off vs. Drug-on patients*						
	**Parietal cortex**					
	Inferior parietal^**^	L	40	−42, −52, 52	14	4.25
	**Temporal cortex**					
	Inferior temporal^***^	L	37	−45, −52, −8	5	5.18
[C-I] (negative faces)						
*A. Drug-on vs. Drug-off patients*						
	*No suprathreshold voxels*					
*B. Drug-off vs. Drug-on patients*						
	*No suprathreshold voxels*					

* *p < 0.001*,

** *p < 0.005*,

*** *p < 0.008, k > 5, n = 10, uncorrected. Cerebral areas are defined by the Automatic Anatomical Labeling. BA, Brodmann areas; C, congruent; H, hemisphere; I, incongruent; k, cluster size (number of voxels); L/R, left/right; MNI, Montreal Neurological Institute; x, y, z, mediolateral, rostrocaudal, and dorsoventral*.

## Discussion

The main finding of this study is the demonstration of a dopaminergic modulation in the rostral anterior cingulate cortex, an area specifically implicated in emotional conflict resolution. During our negative emotional Stroop task, drug-off parkinsonian patients displayed a relative hypoactivation in their rACC compared with healthy controls. No significant difference within the rACC activation was found between the HC and the drug-on patients, suggesting a normalization of the rACC activation deficit after levodopa administration.

Our behavioral results indicated an abnormal negative ES effect in drug-off patients. Controls and drug-on patients presented a negative ES effect, whereas drug-off patients did not, suggesting that drug-off patients were less sensitive to negative emotional interference. Based on the anxiety and depressive literature (Williams and Nulty, 1986; Becker et al., 2001), we would have expected that the drug-off state, which increases anxiety and depressive mood, would be linked to an increased ES effect. We would also have expected that drug-off patients took longer to solve the ES conflict because of the abnormal activation of the rACC and the recruitment of additional brain circuits. Serra-Mestres and Ring (2002) showed that non-depressed parkinsonian patients presented with greater interference to sad words than did HC. However, their patients were tested while on dopaminergic medication and it is difficult to compare their study to ours, due to methodological differences such as the use of another type of ES task and an alternative method for calculating the ES effect. An explanation to understand the diminished emotional interference in our drug-off patients would be that, although most dopamine neurons provide a reward signal, a few dopamine neurons (5–15%) are activated by primary aversive stimuli (Schultz, 2013) This may partially explain why drug-on patients are more sensitive to negative stimuli/distractors than drug-off patients. Another hypothesis could be that Parkinson's disease enhances distractor resistance when patients are in their drug-off state and that dopaminergic medication reinstates susceptibility to distraction (Cools et al., 2010). This could explain the absence of negative distractor-related slowing in drug-off patients, leading to relatively faster responding after distraction than in controls and drug-on patients. Slowing was reinstated by dopaminergic medication, as evidenced by the finding that the patient responses in their drug-on state did not differ from that of controls.

Lastly, another hypothesis to explain the decreased vulnerability to emotional interference could be that the presence of apathy in our drug-off patients enhances their negative emotional blunting, making them less sensitive to negative distractors. Our drug-off patients displayed a tendency to have impaired fearful expression recognition and presented higher scores on the Apathy scale. Numerous studies have demonstrated an impaired recognition of facial expression in Parkinson' disease, especially for negative emotions such as fear, disgust, anger, and sadness (Peron et al., 2012). Clinically, apathy is identified as a reduction of goal-directed behavior because of a lack of feeling, interest, emotional reactivity and motivation (Marin, 1991). Apathetic patients have been shown to have a diminished capacity to process, identify and differentiate between favorable (positive stimuli) and unfavorable (negative stimuli) outcomes and to adjust subsequent behaviors accordingly (Holroyd et al., 2002; Cohen and Ranganath, 2007; Martínez-Horta et al., 2014). Research in the field of apathy has shown impaired emotion recognition and reward processing in the absence of deficits of higher cognition (Martínez-Corral et al., 2010; Lawrence et al., 2011; Robert et al., 2014). In conclusion, apathetic drug-off patients are more impaired than drug-on patients in distinguishing the valence of emotional stimuli and in negative emotion recognition, which could make them less sensitive to negative distractors. It is noteworthy that the ES effect was absent during negative trials in hypodopaminergic patients but present during positive trials, probably because the emotional blunting in PD predominantly affects negative emotions.

The abnormal negative emotional interference in our drug-off patients could be linked with their relative rACC hypoactivation, because this area is specific to emotional conflict and was hypoactive in drug-off patients compared with drug-on patients and HC. Whalen et al. (1998) proposed that rACC activation reflects the successful processing of emotional stimuli in the service of facilitating task performance. The rACC appears to decrease the weighting of irrelevant affective information in the service of optimizing cognitive performance. In healthy volunteers, Bishop et al. (2004) found that the rACC was strongly activated by infrequent threat-related distractors, consistent with a role of this area in responding to unexpected processing conflict caused by salient emotional stimuli. Egner et al. (2008) demonstrated that the rACC was specifically implicated in the resolution of emotional conflict. In our patients, failure to activate the rACC might then reduce the rACC control over negatively salient task-irrelevant emotional stimuli, leading to a reduced recruitment of the normal brain circuitry to overcome emotional conflict and increase the cognitive processing load. In our study, the dorsal ACC, a region implicated in both emotional and non-emotional monitoring, was activated in HC in the Incongruent vs. Congruent trials using all faces, negative faces and positives faces. It was also more activated in drug-on than in drug-off patients, suggesting an abnormal conflict monitoring in hypodopaminergic parkinsonian patients. This relative dorsal ACC hypoactivation might explain why drug-off patients presented with rCCA hypoactivation via probable top-down mechanisms.

The abnormal emotional interference effect and the rACC hypoactivation seen in our drug-off patients were reversed after dopamine administration, suggesting a dopaminergic involvement in emotional conflict. To our knowledge, our study is the first to evoke a specific dopaminergic modulation in the rACC in parkinsonian patients. The ACC hypoactivation in drug-off patients could be directly induced by the degeneration of mesocortical dopaminergic fibers. PD alters the mesocorticolimbic dopaminergic system which consequently impairs the function of the cingulate striatofrontal loop (Czernecki et al., 2002). Drug-on patients did not differ from HC in terms of MRI activation probably because dopatherapy doses were sufficient to restore dopamine function in the mesocortocolimbic pathways in our patients. The ACC receives one of the richest dopaminergic innervations of any cortical area (Gaspar et al., 1989). The source of ACC dopaminergic input comes from cell bodies located in the ventral tegmental area (VTA). Using immunohistochemicals methods, Raghanti et al. (2008) demonstrated a dense dopaminergic innervation in the dorsal ACC (BA 32) in humans. The mesocortical dopaminergic degeneration could then induce a lack of rACC dopaminergic activation. The rACC hypoactivation in drug-off patients could also be explained more indirectly by the degeneration of dopaminergic cells originating from the VTA and projecting to the orbitofrontal cortex (OFC). This denervation could induce a dysfunction in the limbic loop of basal ganglia. This circuit originates in the OFC and the ACC, and projects to the nucleus accumbens and the most rostral portions of the ventral striatum (Oades and Halliday, 1987). The ventral striatum projects in turn to the ventral pallidum, which then projects to the substantia nigra *pars reticulata*, which finally projects to the mediodorsal thalamic nucleus. This circuit is then closed by thalamic projections to the ACC and the medial OFC (Alexander et al., 1986; Hamani et al., 2004). The rACC being a structure involved in the limbic loop of the basal ganglia, the relative rACC hypoactivation demonstrated in our drug-off patients could induce a dysfunction of this circuit.

During the incongruent trials of our ES task, there was conflict between the automatic reading of emotional words (distractors) and the recognition of facial emotion. This situation requires an implicit analysis, which is automatic, subconscious, and rapid. This analysis probably involves the basal ganglia, which are responsible for the automatic execution of learned motor, cognitive, and emotional plans (Marsden, 1982; Alexander et al., 1986). A basal ganglia limbic loop dysfunction would induce an abnormal emotion regulation by cingulate failure to implement control processes over affective distractive stimuli.

Neuropsychiatric fluctuations have been shown to be an important component of levodopa-induced fluctuations (Maricle et al., 1995a). They are related to levodopa dosing (Maricle et al., 1995b). Parkinson's disease behavioral disturbances can be described on a continuous spectrum ranging from hypodopaminergic behaviors (apathy, anxiety, depression and fatigue) to hyperdopaminergic behaviors (mania, impulse control disorder, dopamine dysregulation syndrome, punding and appetitive behaviors) (Ardouin et al., 2009; Lhommée et al., 2012; Rieu et al., 2012; Castrioto et al., 2014). In our patients, acute intake of dopamine replacement therapy improved the Apathy and VAMS scores and was concomitant with normalized ES task behavioral results and rACC activation. This finding could provide a neurobiological basis for the physiopathology of neuropsychiatric fluctuations. During their drug-off state, our patients displayed higher apathy scores and VAMS affective and asthenia subscores, than drug-on patients. We have discussed above how apathy could lead to decreased negative interference in drug-off patients. The VAMS is designed to measure anxiety, mood changes and physical and mental sedation in a non-specific way after the administration of drugs. It is possible than the higher level of anxiety and sedation in our drug-off patients was associated with the rACC hypoactivation through disrupted attentional control over negatively salient task-irrelevant emotional stimuli. This would be consistent with studies addressing anxiety disorders such as Shin et al. (2001) and Kim et al. (2008) who reported that posttraumatic stress disorder patients presented decreased rACC functioning when exposed to situations eliciting negative emotional conflict. In terms of neuroanatomical network, our hypothesis that rACC hypoactivation could explain at least in part the presence of neuropsychiatric fluctuations, is consistent with Thobois et al.'s study (2010) that demonstrated that patients with neuropsychiatric fluctuations had greater mesocorticolimbic dopaminergic denervation than patients without neuropsychiatric fluctuations.

Our drug-off patients exhibited additional areas of cerebral activation not seen in HC and drug-on patients, such as the left superior medial frontal gyrus (BA 8) and the left hippocampus. BA 8 is involved in the management of uncertainty. Increased activation has been shown when subjects experience increasing uncertainty (Volz et al., 2005), which appears to be the case in our drug-off patients. The hippocampus is crucial for long-term episodic memory (Bird and Burgess, 2008) and it has been suggested that it facilitates predictions about upcoming events (Buckner, 2010). It is possible that the limbic basal ganglia loop dysfunction in drug-off patients is compensated by hyperactivation of these two structures. When basal ganglia are inefficient, they cannot execute learned plans in an automatic manner (Marsden, 1982) and drug-off patients rely on compensatory cortical areas based on slow intracortical connections. Compensatory activations have been frequently observed in drug-off patients and impaired patterns of cortical activation tend to normalize with both dopaminergic treatment and subthalamic deep brain stimulation (Jenkins et al., 1992; Limousin et al., 1997).

A number of limitations of our study should be acknowledged. Firstly, we report uncorrected statistics for our fMRI results. The small sample size did not allow for significant brain activation when we applied correction of multiple comparisons. Consequently, our results should be considered preliminary and need to be repeated in a larger population. However, we adopted a more stringent statistical threshold (*p* < 0.001) than would normally be applied for corrected analyses. Each patient served as his own control, meaning that differences between drug-on and drug-off state may be highly significant in relatively small samples. Secondly, we did not include a non-emotional Stroop task for comparison with our ES task. It would have been interesting to use the non-emotional Stroop task described by Egner et al. (2008). Without this task, it is difficult to dissociate the proportion of behavioral slowdown during incongruent trials, due to non-emotional and to emotional interferences. It is also difficult to dissociate the neuroanatomical networks recruited in conflict depending on the nature (emotional or non-emotional) of the conflict. Nevertheless, numerous studies, such as the paper by Egner et al. (2008) have precisely defined which regions are specifically activated in non-emotional and emotional conflict tasks, making the rACC hypoactivation in our drug-off patients specific for emotional conflict resolution.

In conclusion, our study suggests that emotional conflict processes could be dopamine-dependent. Drug-off parkinsonian patients demonstrated a relative underactivity in the affective subdivision of the ACC (rACC) during a negative emotional interference task, which was not seen in the same patients in the drug-on condition, nor in healthy controls. However, our results should be considered preliminary, as further trials with larger sample sizes are required. Rostral ACC hypoactivation could be due to the degeneration of dopaminergic mesocorticolimbic pathways.

In light of the role of the rACC in mediating affective and motivational behaviors (in particular in emotional conflict resolution), we suggest that deficient rACC activation in hypodopaminergic parkinsonian patients might explain their adaptational difficulties in response to negative emotional distractors and promote neuropsychiatric fluctuations such as apathy, anxiety or depression. Our data support the hypothesis stipulating that neuropsychiatric fluctuations have a dopaminergic substrate and reinforces the knowledge that adjustments of dopaminergic medication might be helpful for the treatment of neuropsychiatric fluctuations in Parkinson's disease.

## Funding

This work was funded by the French Parkinson Association and the Grenoble University Hospital.

### Conflict of interest statement

The authors declare that the research was conducted in the absence of any commercial or financial relationships that could be construed as a potential conflict of interest.

## Abbreviations

ACC: anterior cingulate cortex
BA: Brodmann area
BDI: Beck Depression Inventory
BOLD: Blood-Oxygen-Level Dependent
C: congruent trial
CI: confidence interval
DRS: Dementia Rating Scale
ES: emotional Stroop
FAB: Frontal Assessment Battery
FOV: field of view
fMRI: functional MRI
HC: healthy controls
I: incongruent trial
k: cluster size
LEDD: levodopa-equivalent daily dosage
MDD: major depressive disorder
MNI: Montreal Neurological Institute
N: negative facial expression
NMF: non-motor fluctuations
OFC: orbitofrontal cortex
OCD: obsessive-compulsive disorder
P: positive facial expression
PTSD: posttraumatic stress disorder
R1: region 1
R2: region 2
rACC: rostral anterior cingulate cortex
RT: reaction time
SAS: Starkstein Apathy Scale
SD: standard deviation
t: *t*-value (against chance level)
TE: time echo
TR: time repetition
UPDRS: Unified Parkinson's Disease Rating Scale
VAMS: Visual Analog Mood Scale
VTA: Ventral Tegmental Area.
